# Supplementary figures and images for: Breeding Buckwheat for Nutritional Quality in the Czech Republic
Source: Plants (Basel). 2021 Jun 22;10(7):1262. doi: 10.3390/plants10071262 (PMC8309114; doi:10.3390/plants10071262)

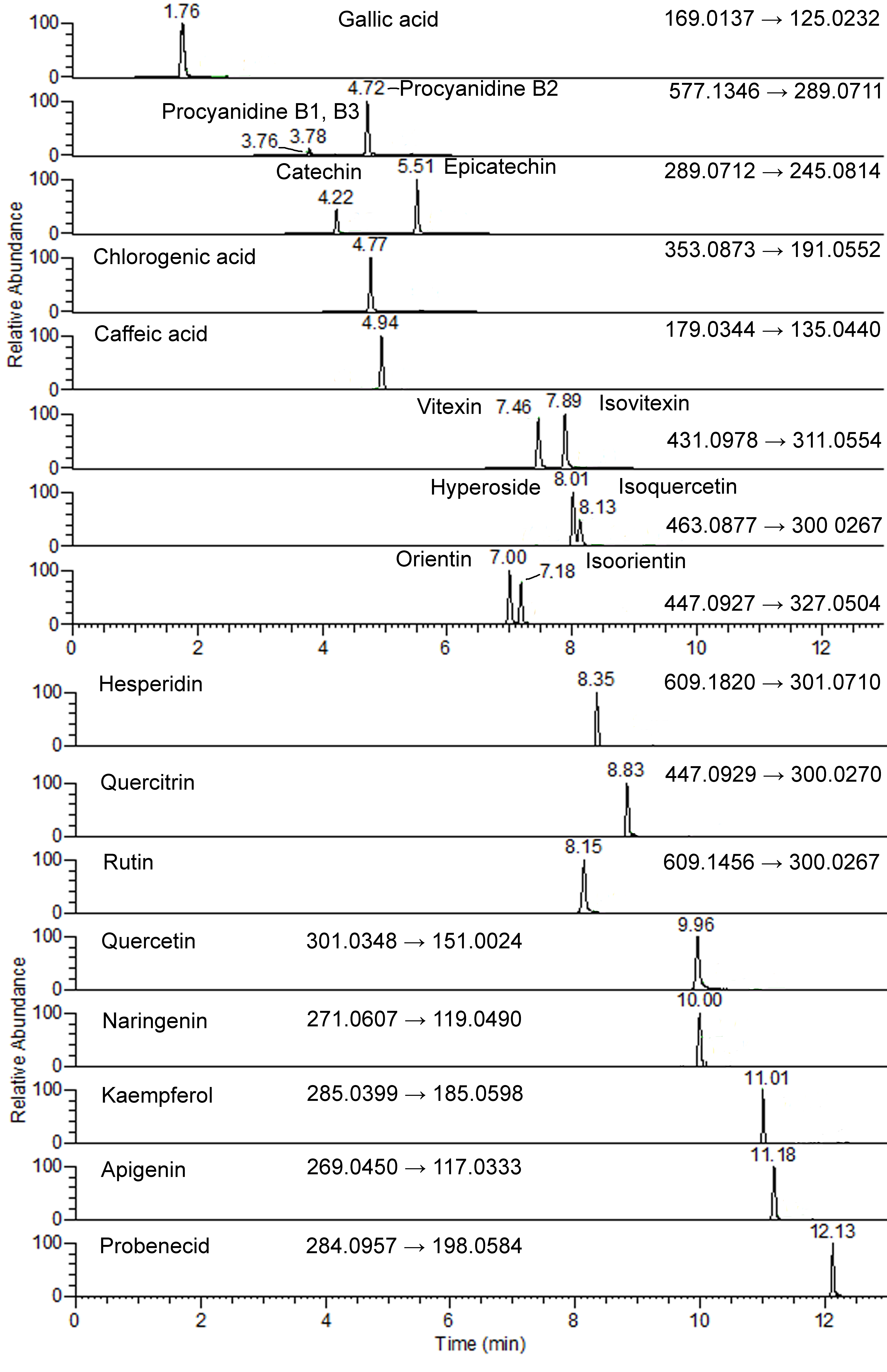

Supplement: Supplementary file 1 [file plants-10-01262-s001.zip › Figure S1.tif]
